# Modulatory Impact of Oxidative Stress on Action Potentials in Pathophysiological States: A Comprehensive Review

**DOI:** 10.3390/antiox13101172

**Published:** 2024-09-26

**Authors:** Chitaranjan Mahapatra, Ravindra Thakkar, Ravinder Kumar

**Affiliations:** 1 Cardiovascular Research Institute, University of California San Francisco, San Francisco, CA 94158, USA; 2 California Institute for Quantitative Biosciences, University of California, Berkeley, CA 94720, USA; 3 Department of Pathology, College of Medicine, University of Tennessee Health Science Center, Memphis, TN 38163, USA

**Keywords:** oxidative stress, APs, biophysics, ion channel, pathophysiology

## Abstract

Oxidative stress, characterized by an imbalance between the production of reactive oxygen species (ROS) and the body’s antioxidant defenses, significantly affects cellular function and viability. It plays a pivotal role in modulating membrane potentials, particularly action potentials (APs), essential for properly functioning excitable cells such as neurons, smooth muscles, pancreatic beta cells, and myocytes. The interaction between oxidative stress and AP dynamics is crucial for understanding the pathophysiology of various conditions, including neurodegenerative diseases, cardiac arrhythmias, and ischemia-reperfusion injuries. This review explores how oxidative stress influences APs, focusing on alterations in ion channel biophysics, gap junction, calcium dynamics, mitochondria, and Interstitial Cells of Cajal functions. By integrating current research, we aim to elucidate how oxidative stress contributes to disease progression and discuss potential therapeutic interventions targeting this interaction.

## 1. Introduction

Oxidative stress occurs when there is an imbalance between the production of ROS and the body’s ability to counteract these harmful molecules with antioxidant defenses. ROS includes singlet oxygen (^1^O_2_), superoxide (O_2_^•−^), hydroxyl ions (^•^OH), and hydrogen peroxide (H_2_O_2_). Nitric oxide (NO) and peroxynitrite (ONOO^−•^), also known as reactive nitrogen species (RNS), are free radicals to oxidize biological tissues. The physiological production of ROS and RNS is carefully regulated by antioxidant systems, including glutathione, catalase, and superoxide dismutase, which efficiently neutralize these reactive intermediates to protect biological systems. The increased levels of ROS can damage essential cellular components such as DNA, proteins, and lipids. Such oxidative damage plays a role in developing various chronic diseases such as cardiovascular diseases, diabetes, cancer, and neurodegenerative diseases (e.g., Alzheimer’s and Parkinson’s). ROS are highly reactive and can alter nucleic acids, leading to mutations and genomic instability [1,2,3,4]. This can initiate or worsen conditions such as cancer, particularly in areas where chronic inflammation and oxidative stress are present, such as the gastrointestinal tract in inflammatory bowel disease [5]. Additionally, oxidative modifications to proteins can impair their function, which is linked to several neurodegenerative diseases. For instance, in Alzheimer’s disease, oxidative stress can cause the aggregation of amyloid-beta peptides, a key feature of the disease [6]. Similarly, in Parkinson’s disease, oxidative damage to dopaminergic neurons accelerates neuronal loss and disease progression [7]. Cardiovascular diseases are also closely associated with oxidative stress. Atherosclerosis, characterized by plaque buildup in arterial walls, is partly driven by the oxidation of low-density lipoprotein (LDL) cholesterol [8]. Oxidized LDL is absorbed by macrophages, leading to foam cell formation and atherosclerotic plaques [8]. Additionally, oxidative stress can cause endothelial dysfunction, a precursor to hypertension and other vascular conditions [9]. This dysfunction reduces the availability of NO, a vital molecule for vascular relaxation, thereby increasing vascular resistance and blood pressure [10]. In chronic inflammatory diseases, oxidative stress results from and contributes to inflammation [11]. For example, rheumatoid arthritis is characterized by ongoing immune cell activation that produces ROS as part of the inflammatory response [12]. These ROS further damage tissues, creating a cycle of inflammation and oxidative stress [13]. In rheumatoid arthritis, oxidative modifications of proteins and lipids exacerbate tissue damage and the autoimmune response [14]. Oxidative stress also plays a role in metabolic disorders such as diabetes mellitus. In diabetes, hyperglycemia leads to excessive ROS production, which in turn causes insulin resistance and beta-cell dysfunction [15]. The oxidative damage to pancreatic beta cells impairs insulin secretion, worsening hyperglycemia and perpetuating metabolic dysregulation [16]. Furthermore, oxidative stress contributes to diabetes complications such as nephropathy, retinopathy, and neuropathy by damaging blood vessels and tissues in various organs [17].

**Figure 1:**
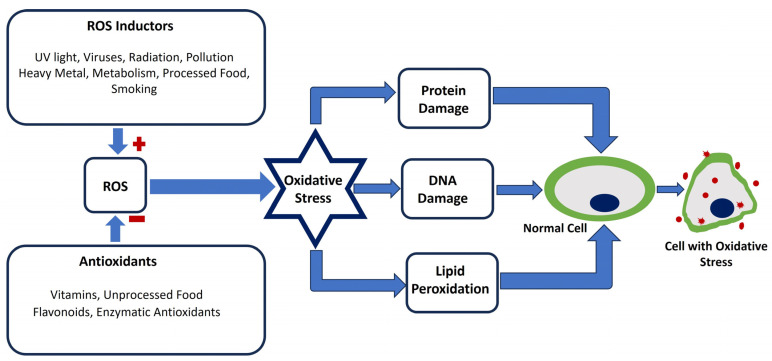
Illustration of the processes by which oxidative stress disrupts normal cells through the induction of reactive oxygen species. The red positive signs indicate ROS enhancers, while the red negative signs indicate ROS inhibitors. Oxidative stress radicals that modulate the shape of the cell are depicted by red stars and circles.

Given its central role in these diverse conditions, oxidative stress is a significant target for therapeutic approaches. Figure 1 illustrates the process by which oxidative stress disrupts normal cells through the induction of reactive oxygen species. Antioxidant therapies aim to restore the balance between ROS production and antioxidant defenses [18]. Research into dietary antioxidants, such as vitamins C and E, and pharmacological agents that enhance the body’s antioxidant systems is ongoing. Additionally, lifestyle changes such as regular exercise and a diet rich in fruits and vegetables can help improve the body’s antioxidant capacity and reduce oxidative stress [19,20]. 

Membrane potential is the electrical difference across a cell membrane, crucial for signal transmission, muscle contraction, and maintaining cell homeostasis. APs are rapid, transient electrical signals that travel along the membranes of excitable cells, playing a fundamental role in communication within the nervous system and between different tissues [21,22,23]. The generation and propagation of AP are crucial for normal physiological processes, including muscle contraction, sensory perception, and cognitive functions [24,25,26]. An AP begins with membrane depolarization when the membrane potential rises to a critical threshold, leading to the opening of voltage-gated sodium (Na^+^) channels and the propagation of the electrical signal. This depolarization process is regulated by various mechanisms, including ion channels that manage ionic flow, gap junctions that enable intercellular electrical connectivity, and calcium (Ca^2+^) dynamics that affect cellular excitability [27]. Mitochondria also play a role by supplying ATP necessary for the activity of ion pumps [28]. At the same time, interstitial cells of Cajal (ICC) are essential for modulating rhythmic depolarization patterns in smooth muscle tissues [29]. The AP generation process begins with depolarization, where sodium (Na^+^) ions enter the excitable cells, causing the membrane potential to become less negative [22]. An excitatory postsynaptic potential is a brief increase in the membrane potential of a postsynaptic neuron, enhancing the likelihood of triggering an AP. The “threshold” for Na^+^ voltage-gated channels is the specific membrane potential needed to activate these channels. Once this threshold is met, a rapid influx of Na^+^ ions occurs, leading to depolarization and the initiation of an AP. During repolarization, potassium (K^+^) voltage-gated channels open, allowing K^+^ ions to flow out of the excitable cells. This outflow counteracts the depolarization by restoring the membrane potential to its resting state, thus quickly returning it to a negative value. A hyperpolarization phase may occur if the membrane potential becomes temporarily more negative than the resting level [26]. Excitable cells can generate various firing patterns, such as burst firing, which consists of rapid clusters of APs, and slow wave (SW) firing, characterized by rhythmic, periodic bursts of APs. Burst firing is often initiated and sustained by T-type Ca^2+^ currents, with Ca^2+^-activated K^+^ currents influencing the bursts’ duration and frequency [30]. Slow wave firing, on the other hand, is mainly driven by persistent Na^+^ currents and low-threshold Ca^2+^ currents, which generate the rhythmic, oscillatory activity associated with this pattern.

**Figure 2:**
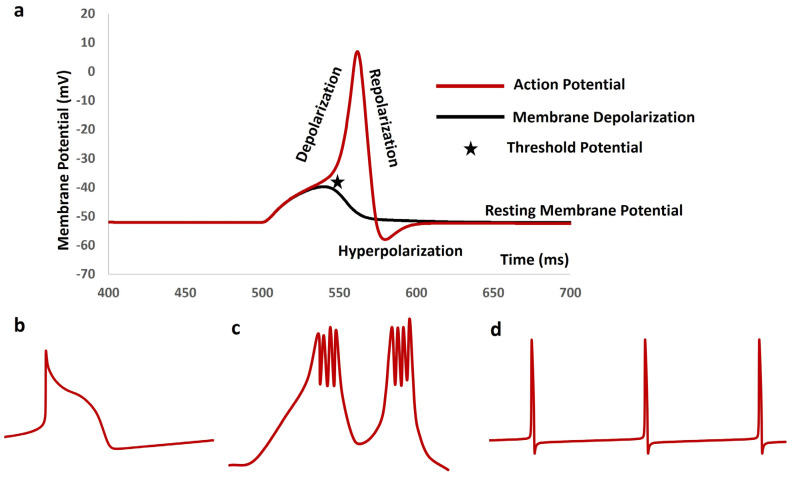
(**a**) Illustration of the simulated membrane depolarization (black solid line), AP (red solid line), depolarization phase, repolarization phase, threshold potential (star mark), and resting membrane potential, which is maintained at −52 mV. (**b**–**d**) show simulated cardiac AP, slow wave with a burst, and series of neuronal Aps, respectively. The *X*-axis represents unscaled time, while the *Y*-axis represents unscaled membrane potential.

Figure 2a is a simulated output that demonstrates the membrane depolarization (black solid line), AP (red solid line), depolarization, repolarization, threshold potential (star mark), and resting membrane potential (RMP). The RMP is maintained at −52 mV. The AP is simulated from the detrusor smooth muscle cell. It is important to note that RMP varies between different tissues, and the duration of the AP can range from a few milliseconds to several seconds depending on the type of excitable cell. Figure 2b–d shows simulated cardiac AP, a slow wave with a burst, and a series of neuronal APs, respectively. The *X*-axis represents unscaled time, while the *Y*-axis represents unscaled membrane potential.

Table 1 lists the types of excitable cells, their RMP values, and AP/SW generation patterns with references. 

However, abnormalities in APs can lead to various pathophysiological conditions, profoundly affecting health. At the core of an AP is the coordinated opening and closing of voltage-gated ion channels, primarily Na⁺ and K⁺ channels [36,37,38]. This orchestrated ion movement generates the rapid depolarization and subsequent repolarization phases characteristic of an AP. In pathological states, these channels’ function or expression disruptions can alter AP characteristics, impacting cellular communication and function [39].

Dysfunctional APs are linked to several neurological conditions [40,41]. In epilepsy, for instance, excessive, synchronous firing of neurons leads to seizures. This hyperexcitability is often due to mutations in ion channels, resulting in prolonged depolarization or insufficient repolarization, thus making neurons more likely to fire inappropriately [42]. Similarly, in multiple sclerosis, demyelination disrupts the efficient propagation of APs, leading to slowed or blocked signal transmission and a range of neurological deficits [43,44]. The heart’s rhythm is tightly regulated by APs generated and propagated through cardiac muscle cells. Pathological changes in ion channels or the cellular environment can lead to arrhythmias, in which the heart beats too quickly, slowly, or irregularly [45]. For example, mutations in Na⁺ or K⁺ channels can cause long QT syndrome, a disorder that prolongs the repolarization phase of the cardiac AP, increasing the risk of sudden cardiac death [46]. APs are integral to the perception of pain and other sensory modalities [47]. In chronic pain conditions, such as neuropathic pain, there is often increased excitability of pain pathways due to changes in ion channel function that lower the threshold for AP generation [48]. This heightened sensitivity can lead to pain perception in response to normally non-painful stimuli (allodynia) or exaggerated pain responses to mildly painful stimuli (hyperalgesia). APs trigger muscle contractions by causing the release of Ca^2+^ within muscle cells [49,50]. Diseases such as myotonia and periodic paralysis involve mutations in ion channels that disrupt normal AP generation or propagation in muscle fibers. These disruptions can lead to symptoms such as muscle stiffness or episodic muscle weakness [51,52]. A range of inherited disorders, collectively termed channelopathies, are caused by mutations in genes encoding ion channels [53]. These disorders illustrate the diverse effects of AP dysregulation, impacting the nervous system, heart, muscles, and other tissues [54]. Examples include cystic fibrosis, where defective chloride (Cl^−^) channels affect multiple organ systems, and various inherited epilepsies and myopathies linked to specific ion channel mutations [55]. Therefore, understanding the mechanisms underlying AP generation and propagation and their alterations in disease is crucial for developing targeted therapies for these conditions. 

Oxidative stress and APs are pivotal in the development of numerous diseases, including neurodegenerative and cardiovascular conditions as well as chronic inflammatory and metabolic disorders. Therefore, understanding how ROS impact AP generation and cause cellular damage is crucial for advancing therapeutic strategies. Recent advances in molecular biology and electrophysiology have provided deeper insights into these processes, offering hope for improved treatments and outcomes for patients with disorders related to oxidative stress and AP dysfunction [56]. Although there is extensive literature on the individual roles of oxidative stress and APs in pathological conditions, comprehensive reviews examining the interplay between these two factors are relatively rare. This gap may be due to the interdisciplinary nature of the subject, requiring knowledge in both biology and physics. However, understanding the interaction between AP biophysics and oxidative stress is essential, highlighting the need for more integrative reviews to bridge basic science and clinical applications. This review aims to deepen our understanding of the interplay between AP biophysics and oxidative stress in cellular and subcellular contexts. It provides experimental evidence showing how oxidative stress disrupts ion channels, gap junctions, calcium dynamics, ICC, and mitochondrial functions at the cellular level, ultimately affecting membrane potential and modulating the generation of APs. This comprehensive understanding can offer valuable insights into physiological mechanisms and guide the development of therapeutic interventions to address various disorders and promote optimal health. 

## 2. Materials and Methods

We extensively searched the MEDLINE database via PubMed [57,58], focusing on English-language articles published at any time. Our goal was to explore the relationships between oxidative stress, reactive oxygen species, ion channels, biophysics, gap junctions, neurotransmitters, neuromodulators, calcium dynamics, and intracellular electrical activities (such as depolarization, hyperpolarization, APs, and slow waves), including both experimental and computational studies. We meticulously screened all relevant studies, excluding non-English articles and those duplicating information from other sources. Priority was given to the most recent and comprehensive manuscripts in cases of overlap. Our selection criteria included original research articles, randomized and non-randomized clinical trials, experimental studies, prospective observational studies, retrospective cohort studies, case-control studies, and review articles on the impact of oxidative stress on ion channels and AP modulation. Each included article was scrutinized, and supplementary references were consulted to ensure comprehensive coverage. Finally, we designed a model diagram to illustrate the key steps in the relationship between oxidative stress and APs.

## 3. Ion Channel Biophysics and Oxidative Stress

Ion channels are essential membrane proteins that control the movement of ions across cell membranes, playing crucial roles in physiological processes such as neuronal signaling, muscle contraction, and hormone release. These channels have diverse structures and functions, with key types including voltage-gated, ligand-gated, and mechanically-gated ion channels [59]. Voltage-gated ion channels, such as those for Na^+^, K^+^, Cl^−^, and Ca^2+^, respond to changes in membrane potential, facilitating the rapid generation and propagation of APs in excitable cells [60,61]. Ligand-gated ion channels, such as nicotinic acetylcholine receptors, are activated by specific neurotransmitters or ligands, resulting in ion flow and cellular responses [62]. Mechanically-gated ion channels, found in sensory neurons, open in response to physical stimuli such as pressure or stretch, converting mechanical signals into electrical ones [63]. The activity of ion channels is intricately regulated through mechanisms such as protein phosphorylation, protein-protein interactions, and changes in intracellular ion concentrations [64]. Protein phosphorylation can alter ion channel function by changing their conformation or membrane localization. Additionally, intracellular ion levels, particularly Ca^2+^, are vital in regulating ion channel activity. Variations in Ca^2+^ concentrations can directly impact ion channel gating or indirectly affect channel function via Ca^2+^-sensitive signaling pathways [65]. In summary, the precise regulation of ion channels is essential for controlling cellular electrical activities, including membrane depolarization, maintenance of RMP, hyperpolarization, and AP generation, emphasizing their critical role in maintaining physiological homeostasis. Oxidative stress can modify ion channels, which are crucial for AP generation and propagation. ROS can oxidize ion channel proteins, altering their gating properties and functionality [66]. Oxidative stress can induce channelopathies through several mechanisms, including direct oxidation, where ROS modify the thiol groups of cysteine residues in ion channels, altering their gating and function, such as in voltage-gated K^+^ channels in cardiac cells, leading to arrhythmias. Additionally, lipid peroxidation alters the membrane environment, impacting ion channel fluidity and function. Nitrosative stress, involving reactive nitrogen species, can nitrosylate ion channels, contributing to conditions such as neuronal hyperexcitability. Indirect effects include changes in channel protein expression, modulation of kinases and phosphatases, and inflammatory responses, all of which further disrupt ion channel function. The following section has elaborated on the effects of oxidative stress on various types of ion channels. 

### 3.1. Voltage-Dependent Ca^2+^ Channels

Voltage-dependent Ca^2+^ channels (VDCCs) are complex structures composed of a central pore-forming α-subunit, which senses voltage changes, along with auxiliary β-, γ-, and α2δ-subunits. VDCCs permit the entry of Ca^2+^ ions into the cell during depolarization, complementing the role of Na^+^ ions in triggering AP generation [67]. Their activation prolongs the depolarization phase and enhances the strength and duration of the AP. At least ten different genes encode the α-subunits in mammals, with the Cav1.2 (L-type Ca^2+^ channel) gene being a prominent one. These channels are subject to redox regulation through the oxidation and nitrosylation of cysteine residues within the α-subunit by ROS and NO. Studies in cardiac myocytes have shown that redox regulation can either enhance or diminish channel function, impacting open time, probability, expression, and trafficking of the channels [68]. While S-nitrosylation can enhance L-type Ca^2+^ currents, tyrosine nitration during oxidative stress typically inhibits them, indicating the type of posttranslational modification critically influences channel activity [69]. ROS can oxidize key amino acids in the L-type Ca^2+^ channel proteins, such as cysteine and methionine residues. This oxidation can lead to structural changes or conformational alterations that impact channel activity and Ca^2+^ ion conduction [70]. Specific cysteines within the extracellular loop of T-type Ca^2+^ channels (Cav3.1) are potential sites for channel gating affected by redox agents [71]. NO often acts by activating soluble guanylate cyclase (sGC), which increases the production of cyclic GMP (cGMP). Elevated cGMP levels can lead to the activation of protein kinases, such as protein kinase G (PKG), which can phosphorylate T-type Ca^2+^ channels and alter their gating properties [72]. Oxidation by external H_2_O_2_ enhances the whole-cell Ca^2+^ currents of P/Q-type Ca^2+^ channels by accelerating channel opening. Both ROS and reactive nitrogen species (RNS) increase the activity of these channels, which may contribute to neurodegenerative processes [66]. 

### 3.2. Sodium (Na^+^) Channels

Voltage-gated Na^+^ channels play a crucial role in the depolarization phase of APs in excitable cells. Oxidative modifications can affect Na^+^ channel inactivation kinetics, leading to prolonged APs and increased cellular excitability, relevant in conditions such as epilepsy and ischemic injury [73]. Agents that oxidize thiol groups, such as thimerosal and 4,4-dithiopyridine, can inhibit Na^+^ currents by shifting the voltage dependence of inactivation toward more hyperpolarized potentials while leaving activation unaffected [66,74]. RNS enhances the activity of inactivation-resistant Na^+^ channels in other neuronal types such as hippocampal neurons and posterior pituitary nerve terminals, likely due to differences in channel subtypes [66]. In cardiac cells, the Na^+^ channel SCN5A (Na_V_1.5) is vital for electrical impulse conduction, and oxidative stress is linked to cardiac arrhythmias. Following myocardial infarction, ROS increases, along with lipoxidation products. Oxidants such as tert-butylhydroperoxide can shift the availability curve (steady-state inactivation curve) of Na^+^ currents to hyperpolarized (more negative) potentials, reducing cardiac Na^+^ current at the activation threshold (threshold potential) [75]. This shift can have significant effects on cardiac function, as even minor changes in the RMP can impact heart rhythm. Additionally, exogenous H_2_O_2_ exposure in the heart has been shown to cause activity early after depolarization and trigger activity by downregulating Na^+^ current [76].

### 3.3. Potassium (K^+^) Channels

Voltage-gated potassium channels (Kv) are crucial for various physiological processes, including muscle contraction, neuronal excitation, regulation of RMP, and modulation of AP shape, duration, and frequency [77]. The activity of Kv channels can be influenced by ROS and RNS through modifications of both the α-subunits and the auxiliary β-subunits. The β-subunits interact with the α-subunits to modulate the rate of channel inactivation. Kv β-subunits can act as sensors of lipid oxidation due to their catalytic activity, reducing oxidized phospholipids during oxidative stress, which can alter the inactivation properties of Kv channels [78]. Additionally, Kv β1 and Kv β2 function as redox enzymes, converting aldehyde to alcohol with Nicotinamide adenine dinucleotide phosphate (NADPH) as a cofactor, linking the cellular redox state to changes in cellular excitability through the Kv β1–Kv β2 complex [79]. Ca^2+^-activated K^+^ Channels (KCa) channels open in response to intracellular Ca^2+^ concentrations and/or depolarization, leading to membrane hyperpolarization and a reduced probability of voltage-dependent Ca^2+^ channel opening [80,81]. KCa channels are classified into three subtypes based on conductance and Ca^2+^ sensitivity: large conductance (BKCa), small conductance (SKCa), and intermediate conductance (IKCa) [82]. The BK channel α-subunit (hSlo) includes seven transmembrane regions. Oxidation of cysteine residues in hSlo1 reduces channel open probability, while oxidation of methionine by chloramine-T increases it [83,84]. In addition, monochloramine enhances BK channel activity and shifts voltage dependence to more negative potentials, an effect blocked by the sulfhydryl alkylating agent N-ethylmaleimide [85]. NO donors potentiate BKCa currents in hypophyseal nerve endings and smooth muscle cells by modifying critical thiol groups [86]. 

ATP-Sensitive K^+^ Channels (K_ATP_) channels are found in smooth muscles, pancreatic β-cells, myocardium, and neurons and play a crucial role in modulating APs, where energy metabolism is closely linked to electrical activities [87]. They consist of four regulatory sulfonylurea subunits (SUR1, SUR2A, or SUR2B) and four ATP-sensitive pore-forming inwardly rectifying potassium channel subunits (Kir6.1 or Kir6.2) [88]. ROS can modulate K_ATP_ activity differently across tissues, reducing activity in cerebral arterioles and coronary arteries while facilitating opening in cardiac myocytes and pancreatic β-cells [89,90]. NO activates K_ATP_ channels through S-nitrosylation of the SUR1 subunit, with mutation of Cys717 reducing NO-induced currents [90]. S-glutathionylation inhibits Kir6.1/SUR2B channels by targeting Cys176, preventing necessary conformational changes for channel gating [91]. Hydrogen sulfide (H_2_S) also targets KATP channels through sulfhydration of cysteine residues in Kir6.1 or SUR, enhancing channel activity, particularly during colonic inflammation [92,93]. The human ether-à-go-go-related gene 1 (hERG1, Kv11.1, KCNH2) encodes the pore-forming subunit of the K^+^ channel responsible for the rapid component of the delayed rectifier current (I_Kr_) in the heart, essential for terminating cardiac APs [94]. Impairment of hERG channel function can lead to long-QT syndrome due to gene mutations, off-target drug effects, or posttranslational modifications [95,96]. Oxidative stress reduces hERG function by decreasing protein levels and accelerating deactivation, primarily through thiol modification of Cys723, with contributions from Cys740 and Cys828 [97,98]. 

### 3.4. Transient Receptor Potential (TRP) Channels

TRP channels are proteins with six transmembrane domains and a pore region between the fifth and sixth domains. They assemble into homo- or heterotetramers, forming nonselective and weakly voltage-sensitive channels [99]. During membrane depolarization, these channels permit the entry of cations, initiating the depolarizing phase of APs. Their activation is crucial for generating APs and modulating cellular excitability, thereby supporting both the baseline electrical state of cells and the rapid changes needed for signal transmission [100,101]. These channels play significant roles in sensory systems, responding to environmental signals such as temperature changes, pH variations, and volatile chemicals. Some TRP channels detect oxidative stress through second messenger production [102], while others are directly influenced by oxidative stress. TRPC3 channels likely interact with signaling molecules within caveolae or lipid rafts, with membrane cholesterol oxidation potentially activating during oxidative stress [103]. TRPM2 is a Ca^2+^-permeable cation channel that also functions as an ADP-ribose hydrolase. It can be activated by H_2_O_2_ either through NAD+ [103] or by direct oxidation [104]. The conversion of NAD+ to ADP-ribose, a potent activator, is likely responsible for H_2_O_2_-induced TRPM2 activation [105,106]. TRPM2 acts as a cellular oxidant sensor, with Ca^2+^ influx via ROS-activated TRPM2 inducing chemokine production in monocytes and exacerbating inflammatory neutrophil infiltration [107]. TRPM6, an epithelial Mg^2+^ channel, is highly expressed in the renal and intestinal cells’ luminal membrane and regulates transepithelial Mg^2+^ transport. TRPM6 and its homologue TRPM7 have ion channel regions combined with serine/threonine kinase domains. RACK1 and REA interact with this domain, inhibiting channel activity in an autophosphorylation-dependent manner [108]. H_2_O_2_ decreases TRPM6 activity, but methionine sulfoxide reductase (MsrB1) can restore it by reducing the oxidation of Met1755, suggesting MsrB1 modulates TRPM6 during oxidative stress [109]. TRPV1 channels are activated by noxious heat, capsaicin, and acidic pH. Various signaling pathways modulate TRPV1, affecting nociceptive neuron excitability. Oxidative stress causes potent, long-lasting TRPV1 sensitization via inter-cysteine disulfide bond formation within its cytoplasmic termini. TRPV1 also regulates NADPH oxidase-mediated ROS generation in microglia [110]. 

### 3.5. Orai Ion Channels

Store-operated calcium entry (SOCE) and Ca^2+^ release-activated Ca^2+^ (CRAC) channels play a pivotal role in modulating APs by regulating intracellular Ca^2+^ levels [111]. When intracellular Ca^2+^ stores, primarily within the ER, are depleted, STIM proteins (STIM1 and STIM2) sense this depletion and activate Orai channels in the plasma membrane, facilitating SOCE [112]. The influx of Ca^2+^ through CRAC channels replenishes intracellular Ca^2+^ levels and sustains prolonged Ca^2+^ signaling, which is critical for various cellular functions including the modulation of APs. Increased intracellular Ca^2+^ concentration can activate calcium-dependent potassium channels, leading to membrane hyperpolarization and affecting the firing rate and pattern of APs [113]. Redox agents can modify both STIM and Orai proteins [114]. Oxidizing agents such as thimerosal and H_2_O_2_ are known to reduce CRAC currents [115]. I_CRAC_ plays a crucial role in T lymphocyte activation, proliferation, and differentiation. During inflammation, lymphocytes encounter highly oxidative environments. Orai1 channels, but not Orai3, are inhibited by H_2_O_2_ oxidation. This difference in redox sensitivity is attributed to an extracellular reactive cysteine present in Orai1 but absent in Orai3 [116]. Consequently, cellular responses to oxidative stress vary depending on the expression of different Orai isoforms. For instance, naive T helper (TH) cells, which have low Orai3 expression, are more redox-sensitive, whereas effector TH cells, which have increased Orai3 expression, exhibit reduced redox sensitivity [117].

### 3.6. P2X2 Receptors

P2X2 receptors, which are ligand-gated ion channels activated by ATP, play a crucial role in modulating APs by influencing synaptic transmission and neuromuscular communication [118]. When ATP binds to P2X2 receptors, it causes the channel to open, allowing the influx of cations such as Na^+^ and Ca^2+^ [119]. This influx depolarizes the membrane, thereby influencing the initiation and propagation of APs. P2X receptors are allosterically modulated by binding with mercury and copper within the receptor’s ectodomain [119]. Mercury, which induces oxidative stress, enhances the activity of P2X2 receptors. Additionally, the activity of P2X2 receptors is modulated by oxidative stress, as evidenced by their potentiation by H_2_O_2_ and the enhancement of their function in the presence of mercury [120]. This redox sensitivity can significantly affect neuronal excitability and synaptic plasticity, potentially altering the patterns of AP firing and transmission under conditions of oxidative stress [121]. Thus, P2X2 receptors serve as key modulators of AP dynamics, integrating extracellular ATP signals and oxidative cues to regulate neuronal and muscular responses. The activation of P2X2 receptors is also potentiated by H_2_O_2_, and this effect is eliminated by the alkylation of Cys430 [122]. 

Table 2 illustrates the role of various ion channels in excitable cell AP/SW generation. 

## 4. Gap Junction and Oxidative Stress

**Figure 3:**
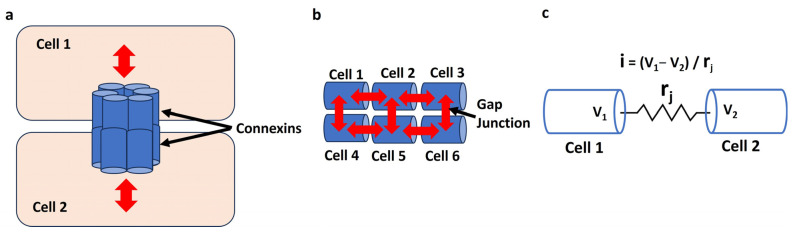
(**a**) shows how connexins from Cell 1 and Cell 2 form a gap junction, enabling signal transfer between them, as indicated by the red bidirectional arrow. (**b**) depicts six cells connected in a linear arrangement through gap junctions (red arrow), illustrating signal transmission along this network. (**c**) is a schematic of the gap junction between Cell 1 and Cell 2, where V_1_ and V_2_ represent their membrane potentials, and r_j_ indicates the gap junction resistance.

Gap junctions are crucial for the electrical properties of excitable cells, such as neurons and cardiac and smooth muscle cells, as they facilitate direct intercellular communication [136]. These specialized membrane channels allow the passage of ions and small molecules between adjacent cells, enabling synchronized electrical activity. By permitting the rapid and coordinated spread of membrane depolarization and APs, gap junctions ensure efficient transmission of electrical signals across cell networks. The permeability and regulatory mechanisms of gap junctions further influence the electrical behavior of excitable cells. Connexins (Cx), the protein subunits that form gap junction channels, can be modified by various factors such as pH, Ca^2+^ concentration, and phosphorylation [137]. These modifications can alter the conductance and selectivity of gap junctions, thereby impacting the intercellular transfer of ions and signaling molecules. For instance, during cardiac ischemia, changes in intracellular conditions can lead to the closure of gap junctions, contributing to arrhythmias. In neurons, dynamic regulation of gap junctions affects synaptic plasticity and can play a role in the development and modulation of neural circuits. Therefore, gap junctions not only enable direct electrical coupling but also integrate various physiological signals to fine-tune the electrical properties of excitable cells [138]. Figure 3a illustrates how connexins from cells 1 and 2 come together to form a gap junction, facilitating the transfer of signals between the two cells. The red bidirectional arrow indicates that each cell can both send and receive signals from the other. Figure 3b demonstrates the connection of six cells in a linear arrangement through gap junctions (red arrow), showing how signals are transmitted along this one-dimensional network. Figure 3c is a schematic representation of the gap junction connection between two cells, Cell 1 and Cell 2. In Figure 3c, V_1_ and V_2_ represent the membrane potentials of Cell 1 and Cell 2, respectively, while r_j_ denotes the gap junction resistance between them.

Oxidative stress, marked by the excessive production of ROS, profoundly affects connexins and gap junctions by disrupting their expression, function, and stability, all of which are essential for cellular communication and the propagation of APs and membrane potentials. Knockdown of Connexin type “Cx43” in cortical astrocytes is reported to increase cell death induced by ROS hydrogen peroxide (H_2_O_2_) [139]. It is shown that a localized oxidative insult to endothelial cells (ECs) propagates through gap junction intercellular communication [140]. Oxidative stress also induces post-translational modifications of connexins, such as phosphorylation, nitrosylation, and oxidation, which can impair gap junction assembly, conductance, and gating. Additionally, oxidative stress promotes the degradation of connexins via proteasomal and lysosomal pathways and can lead to the internalization and disassembly of gap junctions, disrupting intercellular communication. These alterations contribute to various pathological conditions, including cardiovascular diseases, neurodegeneration, and cancer [141,142,143].

## 5. Interstitial Cells of Cajal and Oxidative Stress

**Figure 4:**
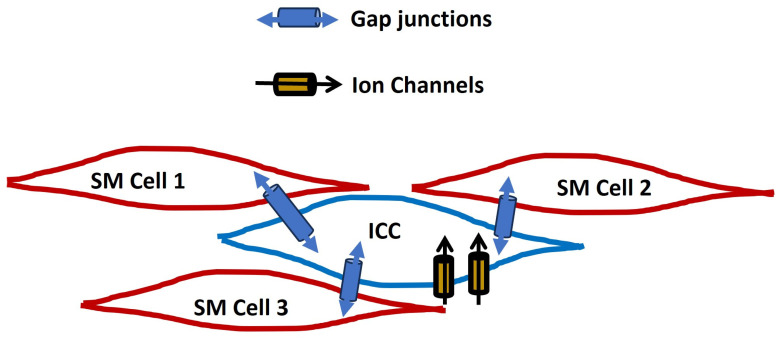
A schematic representation of the ICC cell among smooth muscle cells (SM cells). An ICC cell consists of several ion channels and is connected to neighboring cells via a gap junction.

Interstitial cells of Cajal (ICCs) are well-known for their essential pacemaker role in the gastrointestinal tract, but their presence in other tissues underscores their importance in regulating smooth muscle activity throughout the body. These ICC-like cells contribute to various functions, including urinary flow, vascular tone, airway constriction, reproductive processes, and bile regulation [144]. Although classical ICCs have not been confirmed in the brain, research is investigating ICC-like cells in this context. These cells might affect neural activity, neurovascular coupling, or brain development and repair [145]. ICCs are electrically coupled to neighboring smooth muscle via gap junctions and modulate membrane potential to evoke APs. ICCs generate rhythmic electrical SWs, and the SWs propagate through the smooth muscle layers, coordinating peristalsis and other motility patterns by modulating membrane electrical properties through the regulation of various ion channels. Figure 4 illustrates the ICC cell among smooth muscle cells (SM cells). An ICC cell consists of several ion channels and is connected to neighboring cells via a gap junction.

Two ion channels, ANO1 (also known as TMEM16A) and TRPM7, are of particular importance in the function of ICCs [146]. SWs recorded from ICCs in the stomach and small intestine exhibit distinct phases: an initial rapid depolarization, where the membrane potential rises from −80 to −50 mV up to −25 to 0 mV; a plateau phase, where the membrane potential stays near its peak; and a repolarization phase, where the cell returns to its resting potential [147]. The depolarization and repolarization phases of these waves, driven by the precise opening and closing of these ion channels, create the electrical signals needed for synchronized muscle contractions. However, oxidative stress negatively affects ICCs by increasing ROS production, which leads to cellular damage and altered membrane potential by disturbing ion channel function [148]. TMEM16A ion channel kinetics is modulated by the elevated ROS by the Nox2 NADPH Oxidase (NADPH) oxidase 2 enzyme [149]. The zinc-binding motif of TRPM7 functions as an oxidative stress sensor, regulating the channel’s activity in response to oxidative conditions [150]. In jejunum samples from mice exposed to pro-inflammatory cytokines, Gamma interferon lipopolysaccharide (IFNγ-LPS)-mediated inflammation was found to disrupt the pacemaker function of ICCs without causing ultrastructural or apoptotic changes [150]. This inflammatory state induced oxidative stress through NO synthesized by macrophages and smooth muscle cells, leading to phenotypic changes in ICCs. In patients with neurodegenerative diseases such as Alzheimer’s and Amyotrophic Lateral Sclerosis, increased serum levels of NADPH oxidase 2 (NOX2) and elevated plasma levels of LPS from intestinal Gram-negative bacteria have been observed [151]. During sepsis, IL-17 activates macrophages in the muscle layer, causing structural damage to ICCs due to NOS-induced oxidative stress [152]. It has been proposed that NO synthesis in ICC enhances intracellular Ca^2+^, amplifying inhibitory signals; however, abnormal elevation of intracellular Ca^2^+ can produce cytoplasmic free radicals, increasing cellular damage [153]. Although experimental evidence in ICCs is lacking, NOS overexpression in neurons has been linked to enteropathies such as gastroparesis, achalasia, Hirschsprung’s disease, and diabetic colonic dysfunction [154]. The nitrergic system’s involvement in diabetic gastropathy is marked by reduced active neuronal NOS in the antrum, especially in women, and a decrease in myenteric and nitrergic neurons in the jejunum, ileum, and colon of diabetic rats, although this is not observed in the duodenum [155]. The cyclic guanidine monophosphate (cGMP)-dependent kinase Prkg1, expressed in ICCs as its β isoform (PKG1β), is essential for ICC survival and gastrointestinal motility by modulating NO neurotransmission [156]. ICCs expressing Prkg1 indicate they are key targets of NO released from enteric neurons and play a role in regulating nitrergic signaling [157]. The absence of ICCs and resulting excess NO at the smooth muscle level may explain reduced intestinal motility, indicating that gastrointestinal motility disorders due to ICC depletion are also linked to defective enteric neurotransmission [158]. 

## 6. Calcium Dynamics and Oxidative Stress

**Figure 5:**
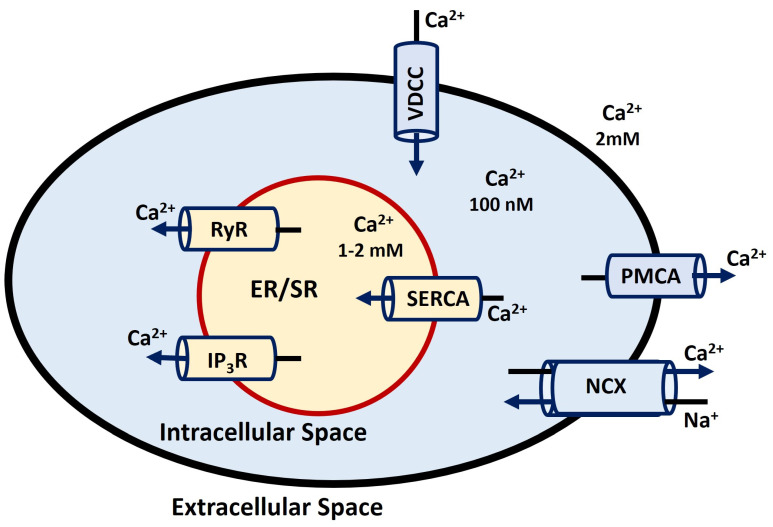
Illustrates Ca^2+^ dynamics processes with all cellular and sub-cellular compartments described in the previous paragraph.

Calcium dynamics and signaling pathways are crucial for numerous cellular processes, including muscle contraction, neurotransmitter release, gene expression, as well as cell proliferation and differentiation [159,160,161,162]. Furthermore, dysregulation of Ca^2+^ signaling pathways has been implicated in numerous diseases, including cancer, neurodegenerative disorders, and cardiovascular diseases [163,164]. Ca^2+^ dynamics play a crucial role in maintaining the RMP by regulating ionic balance, and during membrane depolarization, the influx of Ca^2+^ ions contributes to the depolarizing phase of the AP [165]. Ca^2+^ puffs are localized, transient releases of Ca^2+^ from clusters of IP3 receptors, while calcium sparks are similar localized events but occur at ryanodine receptors in muscle cells. When these localized events spread and propagate across the cell, they form Ca^2+^ waves, coordinating larger-scale cellular responses [166]. Intracellular Ca^2+^ levels are tightly controlled by a delicate balance between Ca^2+^ influx through various channels and Ca^2+^ efflux through pumps and exchangers [167]. Ca^2+^ dynamics involve the careful regulation of Ca^2+^ concentrations within different cellular and sub-cellular compartments, including the endoplasmic/sarcoplasmic reticulum (ER/SR), intracellular space, and extracellular environment. The ER/SR acts as a key Ca^2+^ storage site, where ryanodine receptors (RyR) and inositol 1,4,5-trisphosphate receptors (IP3R) are crucial for releasing Ca^2+^ into the cytoplasm in response to specific triggers [168]. To maintain low cytoplasmic Ca^2+^ levels at rest, the plasma membrane Ca^2+^-ATPase (PMCA) and sodium-calcium exchanger (NCX) are vital in extruding Ca^2+^ from the cell [169]. VDCC facilitates the influx of Ca^2+^ from the extracellular space during membrane depolarization, contributing to the rise in intracellular Ca^2+^. The sarco/endoplasmic reticulum Ca^2+^-ATPase (SERCA) pump then actively transports Ca^2+^ back into the ER/SR, helping to restore intracellular Ca^2+^ levels to baseline and ensuring proper cellular function [170]. Figure 5 illustrates Ca^2+^ dynamics processes with all cellular and sub-cellular compartments. The value of concentrations is borrowed from [171]. 

Oxidants such as superoxide and hydrogen peroxide can significantly inhibit Ca^2+^ transport by SERCA pumps in smooth muscle cells [172]. This inhibition occurs through the oxidation of SERCA’s sulfhydryl groups and direct attacks on its ATP binding site. Thiol-oxidizing compounds and ROS inhibit the Ca^2+^ pumping activity of SERCA, whereas reducing agents such as dithiothreitol and glutathione (GSH) enhance SERCA activity [173]. ROS modulates RyR activity by oxidizing redox-sensitive thiol groups. The oxidation of these thiols enhances channel activity, increases SR Ca^2+^ leak, and elevates Ca^2+^ spark frequency [174,175]. Exogenous oxidants such as thimerosal, t-butylhydroperoxide, and diamide can enhance IP3R-mediated Ca^2+^ release. For example, thimerosal is believed to increase the sensitivity of IP3R to IP3 by oxidizing cysteine residues, thereby stabilizing the receptor in an active conformation [176].

The PMCA is highly susceptible to ROS such as H_2_O_2_, peroxynitrite (ONOO^−•^), and hydroxyl radicals (^•^OH). These ROS can oxidize key amino acids, especially cysteine and methionine residues, within the PMCA. Such oxidative changes can impair the pump’s function, decreasing its ability to effectively remove Ca^2^ from the cytoplasm to the extracellular space [177]. The NCX function is impacted by ROS such as H_2_O_2_, superoxide anions (O_2_^•−^), and peroxynitrite (ONOO^−•^). These ROS can induce oxidative changes in critical amino acids, particularly cysteine and methionine, within the NCX protein, resulting in functional alterations. Such oxidative damage can compromise the exchanger’s efficiency in removing Ca^2+^ from the cell in exchange for sodium ions, potentially causing an increase in intracellular Ca^2+^ levels [178]. Moreover, mitochondria are also central to Ca^2+^ homeostasis; they buffer intracellular Ca^2+^ levels by uptaking and releasing Ca^2^⁺ ions.

## 7. Mitochondria and Oxidative Stress

Mitochondria are essential organelles within cells, primarily recognized for their role in generating ATP through the process of oxidative phosphorylation, which is vital for energy production, cellular metabolism, and the regulation of programmed cell death (apoptosis). Within the mitochondria, the electron transport chain (ETC) consists of a series of protein complexes that transfer electrons, resulting in the formation of a proton gradient across the inner mitochondrial membrane [175]. This electrochemical gradient, known as the proton motive force (PMF), powers ATP synthesis through ATP synthase, with the mitochondrial membrane potential typically ranging from −150 to −180 mV, significantly higher than the potential across the plasma membrane [176,177]. Mitochondria contain various ion channels, including the Mitochondrial Calcium Uniporter, Voltage-Dependent Anion Channel, Mitochondrial Permeability Transition Pore, and Mitochondrial Potassium Channels [178]. These channels are essential for ATP production by maintaining membrane potential and regulating ion and Ca^2^⁺ homeostasis. ATP produced by mitochondria powers ion pumps such as the sodium-potassium ATPase, which sustains ion gradients and supports RMP and AP resetting. Additionally, the release of Ca^2^⁺ from mitochondria can modulate ion channels, thereby affecting membrane potential and cellular excitability [179]. Mitochondria are also a significant source of ROS, which are by-products of oxidative phosphorylation. While low levels of ROS act as signaling molecules, excessive ROS can damage cellular components, including ion channels [180]. This oxidative stress can alter ion channel function, affecting membrane potential and cell excitability [181,182,183]. Furthermore, mitochondria modulate ion channels both indirectly, through the production of metabolic intermediates, and directly, via interactions with mitochondrial proteins [184]. For instance, low ATP levels in mitochondria can activate ATP-sensitive potassium (K⁺) channels, leading to membrane hyperpolarization, reduced excitability, and protection during metabolic stress [185]. In neurons, mitochondria protect against excitotoxicity, a condition where excessive Ca^2^⁺ influx leads to cell death. By buffering Ca^2^⁺, mitochondria help prevent prolonged depolarization and excitotoxic damage [186]. Lastly, mitochondria are highly dynamic and can move within cells to areas of high energy demand, such as synaptic terminals in neurons. This strategic positioning ensures a ready supply of ATP, supporting continuous electrical activity and rapid recovery after APs [187]. Excessive ROS production can cause oxidative stress, damage mitochondrial components, disrupt ATP production, and potentially alter membrane potential, which can impair the cell’s ability to generate APs. 

**Figure 6:**
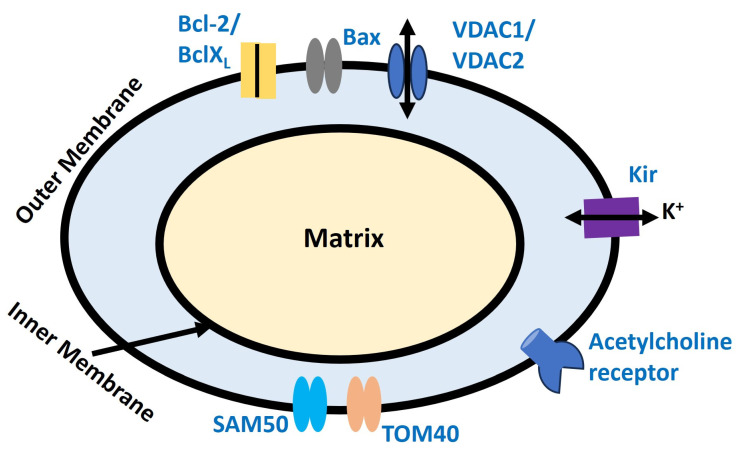
Illustrates protein-mediating ion fluxes in the outer mitochondria membrane described in the previous paragraph. The putative channel acetylcholine receptor is also illustrated.

Figure 6 illustrates the key ion channel components on the outer mitochondrial membrane that are affected by ROS, particularly H_2_O_2_-induced oxidative stress. SAM50 and TOM40 are essential for importing proteins into the mitochondria, with TOM40 forming a channel that also permits ion flow across the outer membrane. SAM50 aids in assembling this complex, ensuring proper channel function [188]. Acetylcholine receptors on the outer mitochondrial membrane form channels that regulate ion flow, influencing mitochondrial function [189]. Bcl-2 and BclX_L_ inhibit apoptosis by regulating ion flow and preventing pro-apoptotic factor release, while Bax promotes apoptosis by forming membrane pores [190]. VDAC1 and VDAC2 at the outer mitochondrial membrane form channels that regulate the flow of ions and metabolites, crucial for cellular energy and signaling [191]. K_IR_ channels at the outer mitochondrial membrane control K⁺ flow, vital for maintaining membrane potential [192]. H_2_O_2_-induced oxidative stress can adversely affect these proteins, impairing their roles in protein import and membrane assembly, which subsequently disrupts mitochondrial function and integrity [193,194,195,196].

## 8. The Model of Oxidative Stress Impact on AP

**Figure 7:**
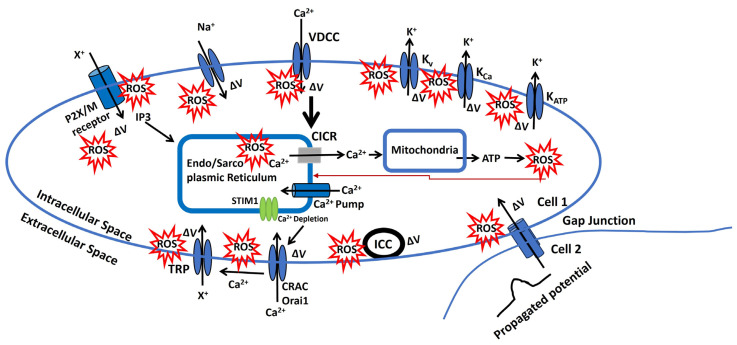
A schematic diagram of the representation of the redox modulation on membrane potential via several pathways that can modulate the AP parameter and cellular excitability.

Figure 7 illustrates a representation of the redox modulation on membrane potential via several pathways, which can modulate the AP parameter and cellular excitability. Nevertheless, the pathophysiology of each step for oxidative stress has not been thoroughly investigated. Instead, these findings are based on the analysis previously mentioned that has been experimentally validated. The ROS is in red star symbol, and the ΔV is known as a change in membrane potential.

1. Endo/Sarcoplasmic Ca^2^⁺ is sourced from the endoplasmic/sarcoplasmic reticulum (ER/SR), an intracellular reservoir. Ca^2^⁺ ions are transported from this storage site to the sarcoplasm via Ca^2^⁺ channels, which are regulated by intracellular agents. Ca^2^⁺ is replenished in the ER/SR by a pump powered by ATP. An increase in the Ca^2+^ concentration near the ER/SR triggers further release of Ca^2+^, which is called the calcium-induced calcium release (CICR) [197]. ROS can influence various factors affecting the filling or release of Ca^2^⁺ in/from the ER/SR. Additionally, Ca^2^⁺ modulates the release of ATP and ROS from mitochondria, and the ROS released can negatively impact the Endo/Sarcoplasmic Ca^2^⁺ dynamics. The red arrow indicates this negative feedback loop from mitochondria to the Endo/Sarcoplasmic reticulum.
2. There is a potential increase in the concentration of a diffusible second messenger, which links the surface membrane to the release of intracellular Ca^2+^. This process primarily involves the activation of purinergic receptors (P2X) or M3 muscarinic receptors. Upon activation, these receptors initiate a series of membrane-bound processes that lead to the production of inositol trisphosphate (IP3). IP3, in turn, can influence Ca^2+^ dynamics as previously described [198]. Changes in the sensitivity or effectiveness of this mechanism can significantly impact the release of intracellular Ca^2+^. ATP may bind to the purinergic receptor (P2X/M), opening a non-specific cation channel that allows the influx of positive ions (X^+^), leading to an increase in membrane potential. This depolarization, modulated by ROS, can open L-type Ca^2+^ channels, facilitate Ca^2+^ influx, and trigger APs.
3. The membrane potential can be transmitted from cell 2 to cell 1 through gap junctions, as some excitable cells function as a syncytium. Moreover, the activation of pacemaking interstitial cells of Cajal (ICC) can also induce an increase in membrane potential. ROS can modulate both gap junction and ICC internal mechanisms, and the resulting depolarization can trigger APs.
4. The voltage-gated and Ca^2+^-activated K^+^ ion channels (K_v_, K_Ca_, and K_ATP_) shown in Figure 7 facilitate the flow of K^+^ from the intracellular to the extracellular space, leading to hyperpolarization. However, the modulating effects of ROS compromise these ion channel mechanisms, resulting in abnormal AP generation. Conversely, VDCC (L-type, T-type, and P/Q type) and voltage-gated Na^+^ channels allow the influx of Ca^2+^ and Na^+^ ions, depolarizing the membrane. ROS also affects these ion channels, contributing to abnormal AP generation.
5. CRAC channels are activated by intracellular depletion mediated by STIM1 and STIM2, allowing an influx of Ca^2+^ that depolarizes the membrane. Ca^2+^, along with other stimuli, can also activate various TRP ion channels, permitting the influx of cations (X^+^) and further depolarizing the membrane to generate APs. Additionally, ROS influences these ion channels, leading to abnormal AP generation.

## 9. Clinical Implications and Future Directions

Understanding how oxidative stress affects ion channels, membrane potential, and AP generation is of great clinical significance. Oxidative stress plays a key role in the development of various pathological conditions, including neurodegenerative disorders such as Alzheimer’s and Parkinson’s diseases, cardiovascular diseases, diabetes, and certain types of cancer. Since ion channels are essential for the proper functioning of neurons, muscles, and other excitable cells, any dysfunction caused by oxidative stress can have serious clinical repercussions. Future research should aim to clarify the complex interactions between oxidative stress and ion channels, enabling the discovery of novel therapeutic strategies to counteract the adverse effects of APs. By enhancing our understanding of the connection between oxidative stress and ion channel biophysics and by translating these insights into clinical practice, we can improve the diagnosis, treatment, and prevention of a wide range of diseases linked to oxidative stress. To fully leverage the insights gained from studying oxidative stress and its impact on ion channel biophysics, several future directions should be pursued:

- **Advanced imaging techniques**: Development of more sensitive and specific fluorescent probes and imaging techniques to measure real-time changes in membrane potential, ROS levels, and ion channel activity in live cells and tissues [199]. This will enhance our understanding of the spatial and temporal dynamics of oxidative stress.
- **High-throughput screening**: Implementing high-throughput screening methods to identify compounds that can protect against oxidative stress-induced ion channel dysfunction [200]. This approach can accelerate the discovery of new therapeutic agents.
- **Integrative multi-omics approaches**: Combining genomics, proteomics, and metabolomics with electrophysiological data to construct comprehensive models of how oxidative stress impacts ion channel function and cellular excitability [201]. This holistic view can uncover new regulatory mechanisms and potential drug targets.
- **Personalized medicine**: Investigating individual variability in oxidative stress responses and ion channel function to develop personalized therapeutic strategies [202]. Genetic and epigenetic factors that influence susceptibility to oxidative stress and ion channel modifications should be identified.
- **Animal models and clinical trials**: Utilizing animal models to study the in vivo relevance of findings from cellular and molecular studies. Translating these findings into clinical trials to evaluate the efficacy of targeted therapies in mitigating the effects of oxidative stress in human diseases [203].
- **Novel therapeutics**: Developing novel antioxidants, ion channel modulators, and gene therapies to specifically address the ion channel dysfunctions caused by oxidative stress [204]. Combination therapies that target multiple pathways involved in oxidative stress responses could prove particularly effective.
- **Computational electrophysiology**: This approach involves using mathematical models to simulate the electrical behavior of cells and tissues. By incorporating data on oxidative stress, such as altered ion channel conductance or gating properties, computational models can predict the impact on membrane potential and AP generation [205]. These simulations can help identify potential therapeutic targets for mitigating the effects of oxidative stress on neuronal and cardiac function.

## 10. Conclusions

This review is crucial in addressing the gap in our understanding of the intricate relationship between oxidative stress and AP modulation in excitable cells. Although significant research has explored the effects of oxidative stress on cellular functions, there remains a lack of comprehensive analyses that link these findings with the biophysics of ion channels and AP dynamics. By integrating these aspects, our review offers a cohesive perspective that connects foundational science with clinical implications, providing insights that could inform the development of targeted therapies for diseases driven by oxidative stress. The review underscores the significant impact of oxidative stress on AP modulation, highlighting its central role in the pathogenesis of various diseases. The complex interaction between ROS and ion channel function emerges as a key mechanism through which oxidative stress disrupts cellular stability. These disruptions in ion channel activity, calcium signaling, mitochondrial function, and intercellular communication via gap junctions contribute to the altered AP generation and propagation seen in neurodegenerative, cardiovascular, metabolic, and inflammatory disorders. The evidence presented emphasizes the importance of ion channels as potential therapeutic targets. Oxidative stress-induced changes—whether via direct oxidation, nitrosylation, or other post-translational modifications—can significantly affect ion channel function, leading to abnormal AP dynamics. A deeper understanding of these molecular changes is essential for developing targeted treatments that restore normal ion channel function and counteract the detrimental effects of oxidative stress. This approach offers promising prospects for treating conditions where oxidative stress is a key driver of disease progression. Moreover, this review highlights the importance of combining advanced experimental techniques with computational models to decipher the complex interactions between oxidative stress and APs. Such interdisciplinary efforts are crucial for deepening our understanding of the underlying molecular mechanisms and for identifying innovative therapeutic strategies. These insights will guide future research, paving the way for novel treatments that tackle the root causes of oxidative stress-related diseases.

In conclusion, research into the modulation of APs by oxidative stress is of critical importance for human health. Ongoing investigations in this area are vital for translating fundamental scientific discoveries into effective clinical treatments. By focusing on the molecular mechanisms through which oxidative stress affects APs, it is possible to develop therapies that improve patient outcomes and alleviate the burden of chronic diseases, ultimately enhancing the quality of life for those affected. This review provides a robust foundation for future research, promoting the development of innovative therapeutic approaches to address the complex challenges posed by oxidative stress in disease progression.

## Figures and Tables

**Table 1:** Values of RMP and types of electrical properties generated in major excitable cells.

Cell Type	RMP (mV)	AP/SW	Reference
Smooth Muscle	−45 to −65	AP/SW	[31]
Cardiac Muscle	−80 to −90	AP	[32]
Skeletal Muscle	−65 to −91	AP	[33]
Neuronal Cell	−60 to −70	AP	[34]
Pancreatic beta cells	−60 to −70	SW	[35]

**Table 2:** Modulating effects of oxidative stress on ion channels for AP/SW generations.

Type of Oxidative Stress	Affected Ion Channels	Mechanism	Effect on AP/SW	Associated Pathological Disorders	Reference
Direct Oxidation	Voltage-gated K^+^ channels (Kv)	Oxidation of thiol groups on cysteine residues alters gating and ion selectivity	Prolonged repolarization, leading to extended AP	Cardiac arrhythmias (e.g., Long QT syndrome), Parkinson’s, Alzheimer’s	[123]
Direct Oxidation	Voltage-gated Na^+^ channels (Nav)	Modification of channel gating, leading to altered excitability	Increased AP amplitude and duration, leading to hyperexcitability	Epilepsy, Neurodegenerative diseases, Chronic pain syndromes	[124]
Lipid Peroxidation	Various membrane-bound ion channels	Disruption of membrane fluidity and integrity, affecting ion channel function	Altered membrane potential, leading to impaired AP propagation	Atherosclerosis, Stroke	[125]
Nitrosative Stress	Voltage-gated Na^+^ channels (Nav)	Nitrosylation leads to altered channel function and increased neuronal excitability	Increased excitability, causing repetitive firing of APs	Epilepsy, Migraine, ALS	[126]
Nitrosative Stress	Voltage-gated Ca^2+^ channels (Cav)	Nitrosylation causing dysregulation of Ca^2+^ homeostasis	Prolonged depolarization phase, leading to excessive Ca^2+^ influx	Neurodegenerative diseases, Cardiac arrhythmias	[127]
Indirect Effects (Inflammation)	Multiple ion channels (Kv, Nav, Cav)	Inflammatory cytokines alter channel expression, kinase/phosphatase modulation affecting channel function	Disrupted AP duration and frequency	Diabetes, Hypertension, Autoimmune disorders (e.g., Multiple Sclerosis)	[128]
Indirect Effects (Kinase/Phosphatase Modulation)	Voltage- K^+^ channels, Ca^2+^ channels	Altered phosphorylation state of ion channels due to disrupted kinase/phosphatase activity	Abnormal AP propagation leading to arrhythmias or seizure activity	Cardiac arrhythmias, Epilepsy, Chronic pain, Ischemic heart disease	[129]
Oxidative Stress-Induced Channel Trafficking Disruption	Multiple ion channels (Kv, Nav)	ROS affects the trafficking of ion channels to the cell membrane, leading to reduced channel availability	Reduced AP initiation and propagation	Cystic Fibrosis, Myotonia	[130]
Direct Oxidation	TRPM channels	Oxidation of critical cysteine residues, affecting channel gating	Altered ion influx, leading to changes in cellular excitability and AP generation	Pain, Inflammation, Neurodegenerative diseases	[131]
Direct Oxidation	Orai (CRAC) channels	ROS-induced conformational changes affecting channel opening	Impaired calcium entry, leading to altered AP and cellular signaling	Immune dysfunction, Autoimmune disorders	[132]
Direct Oxidation	P2X purinergic receptors	Oxidative modification altering ATP binding and receptor activation	Enhanced or reduced receptor activation, leading to changes in excitability and synaptic transmission	Chronic pain, Inflammation, Neurological disorders	[133]
Oxidative Modulation	BK/SK Ca^2+^-activated K^+^ channels	ROS-mediated modulation affecting channel activity	Altered repolarization phase, affecting AP duration and neuronal excitability	Hypertension, Epilepsy, Muscle dysfunction	[134]
Oxidative Stress	TMEM16A/ANO1 channels	ROS-induced modifications affecting channel gate and chloride conductance	Disrupted membrane potential, leading to altered AP propagation and smooth muscle function	Asthma, Hypertension, Cystic Fibrosis	[135]
